# Supplementary material for: First Synthesis of (−)-Altenuene-D3 Suitable as Internal Standard for Isotope Dilution Mass Spectrometry
Source: Molecules. 2019 Dec 12;24(24):4563. doi: 10.3390/molecules24244563 (PMC6943508; doi:10.3390/molecules24244563)
Supplement: Supplementary file 1 [file molecules-24-04563-s001.pdf]

Article

First Synthesis of (–)-Altenuene-D3 suitable as Internal Standard for Isotope Dilution Mass Spectrometry

Michael A. Sebald [1]*, Julian Gebauer [2], Thomas Sommerfeld [3] and Matthias Koch [3]

[1] HPC Standards GmbH, Am Wieseneck 7, D-04451 Borsdorf, Germany; sebald@hpc-standards.com
[2] AnalytiCon Discovery GmbH, Hermannswerder Haus 17, D-14473 Potsdam, Germany; J.Gebauer@ac-discovery.com
[3] Bundesanstalt für Materialforschung und –prüfung, Abteilung Analytische Chemie, Referenzmaterialien, Richard-Willstätter-Strasse 11, D-12489 Berlin-Adlershof, Germany; matthias.koch@bam.de
* Correspondence: sebald@hpc-standards.com; Tel.: +49 (0) 34291 / 33 72 36

Content

^{1}H- and ^{13}C-NMR spectra of all new synthesized compounds. Additional IR- and UV/Vis-spectra of *(-)*-Altenuene-D_3 (**1b**) and native *(-)*-Altenuene (**1a**).

Materials and Methods

NMR spectra were recorded either on a *Varian Mecury Plus 300* (300.8 MHz), *Varian Mecury Plus 400* (399.95 MHz) or a *Bruker Avance III HD* (400.13 MHz) spectrometer with the compounds being dissolved in deuterated solvents. The spectra were processed and the images created with *MestReNova 10* and *MestReNova 14*. The signals were referenced to the solvent signals ($CDCl_3$: ^{1}H-NMR δ = 7.26 ppm, ^{13}C-NMR δ = 77.16 ppm; DMSO-D_6: ^{1}H-NMR δ = 2.50 ppm, ^{13}C-NMR δ = 39.52 ppm).

IR spectra were recorded with a *Bruker Tensor 27* FT-IR spectrometre operated by *OPUS v6.5*. UV/Vis spectra were measured with a V-670 UV-Vis/NIR spectrophotometr by *JASCO*. Visualization of both, IR and UV/Vis spectra, was conducted with *Origin 2017*.

*(2S,3S,4aR,8aR)-7-bromo-2,3,4a,5-tetrahydro-2,3-dimethoxy-2,3-dimethylbenzo[b][1,4]dioxin-6(8aH)-one (**10b**)*

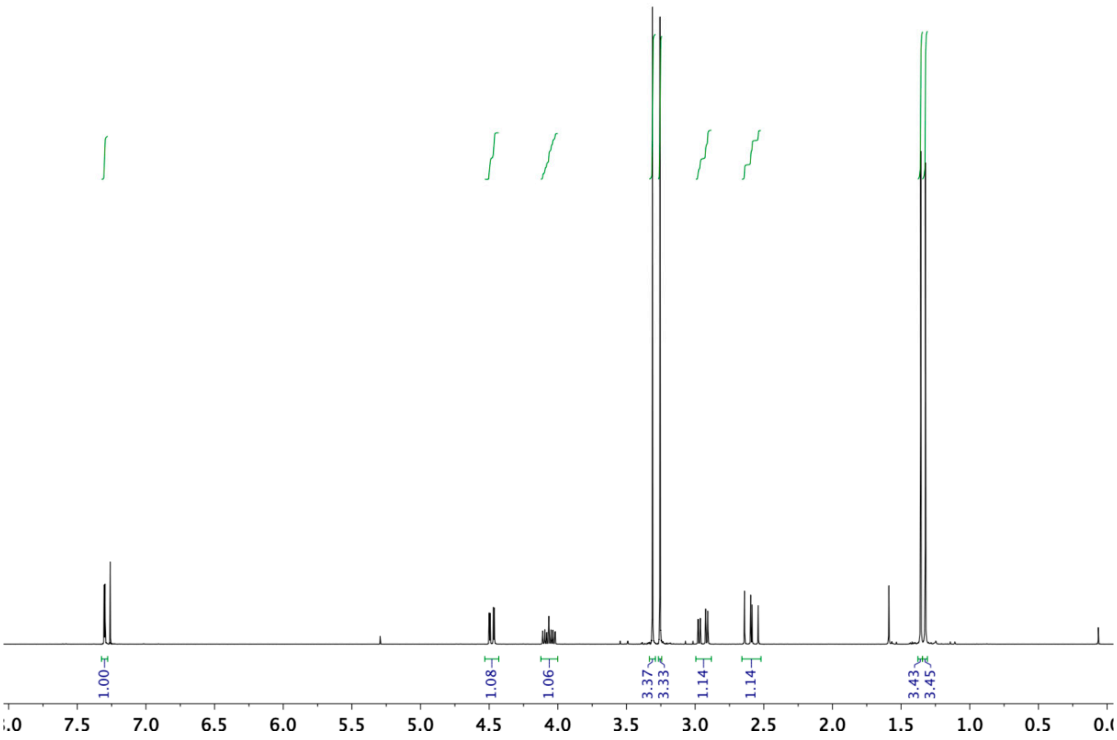

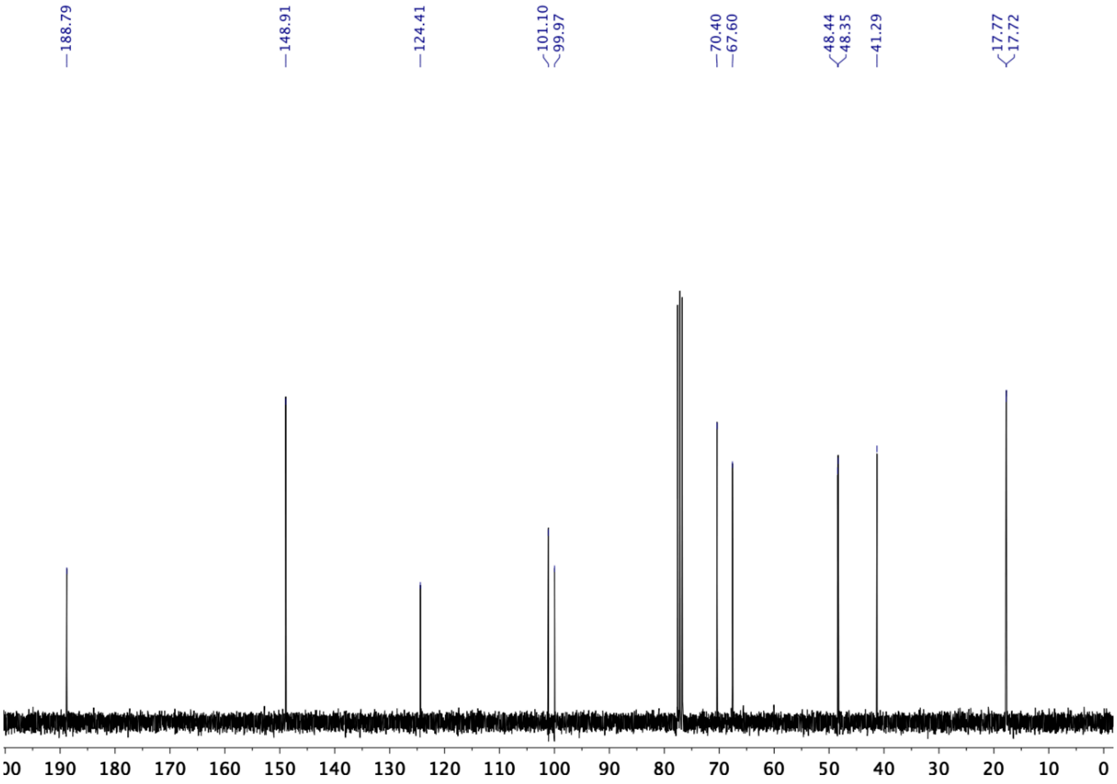

*(2S,3S,4aR,6R,8aR)-7-bromo-2,3,4a,5,6,8a-hexahydro-2,3-dimethoxy-2,3,6-trimethylbenzo[b]-[1,4]dioxin-6-ol (**5b**)*

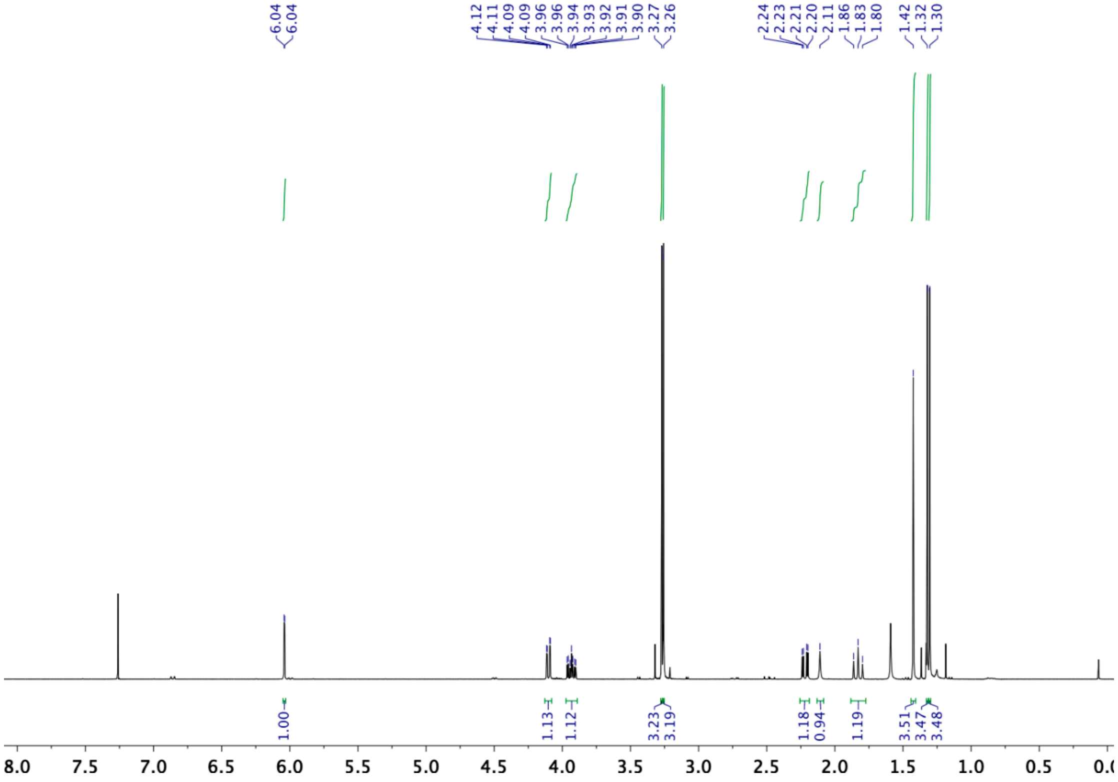

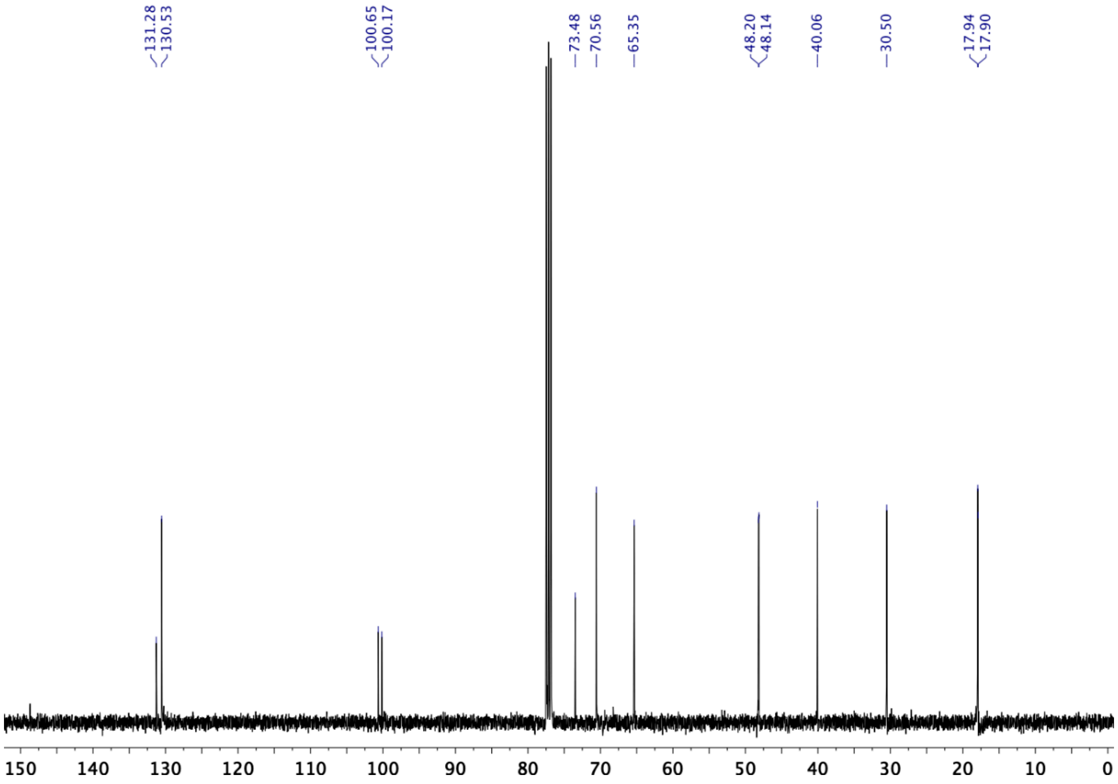

5-hydroxy-7-methoxy-2,2-dimethyl-4H-benzo[d][1,3]dioxin-4-one-D_3 (**7**)

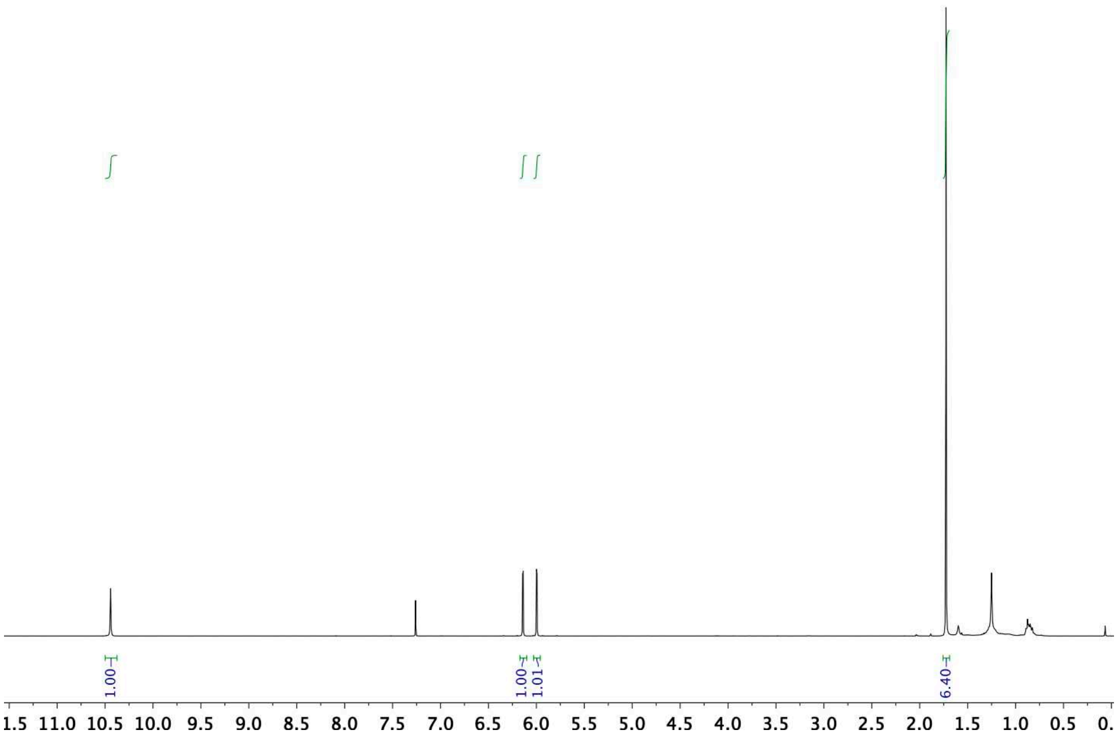

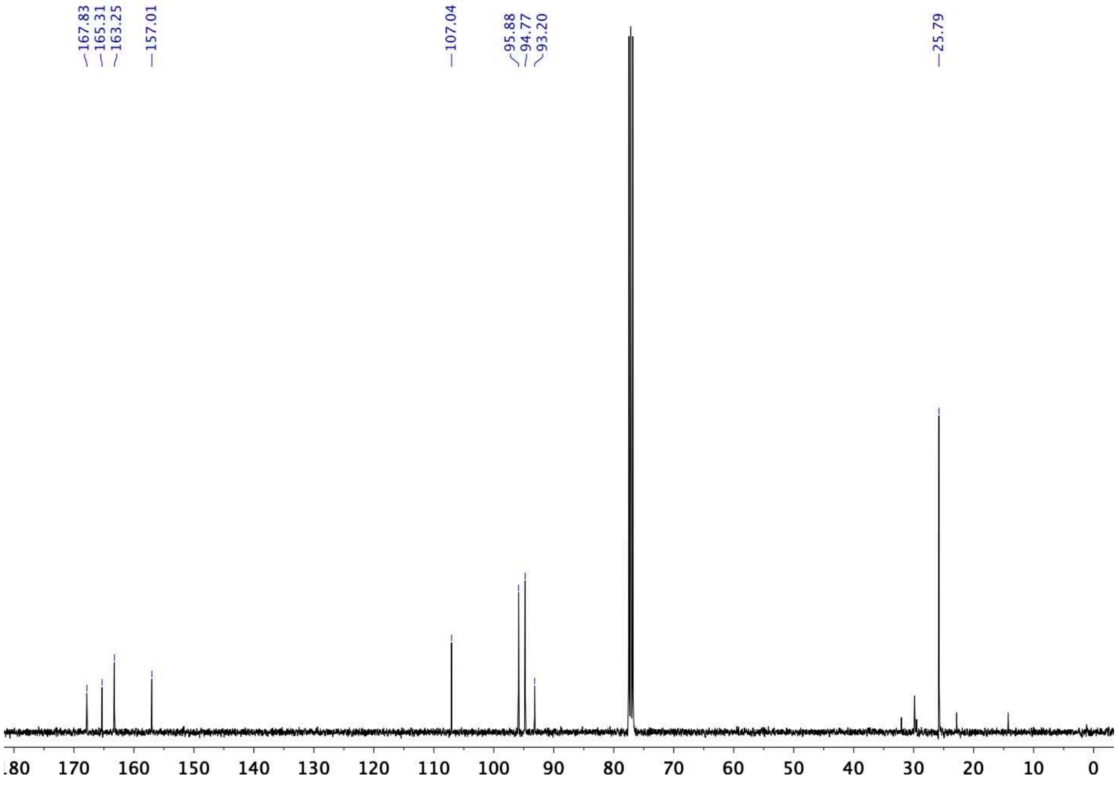

7-methoxy-2,2-dimethyl-4-oxo-4H-benzo[d][1,3]dioxin-5-yl trifluoromethanesulfonate-D$_3$

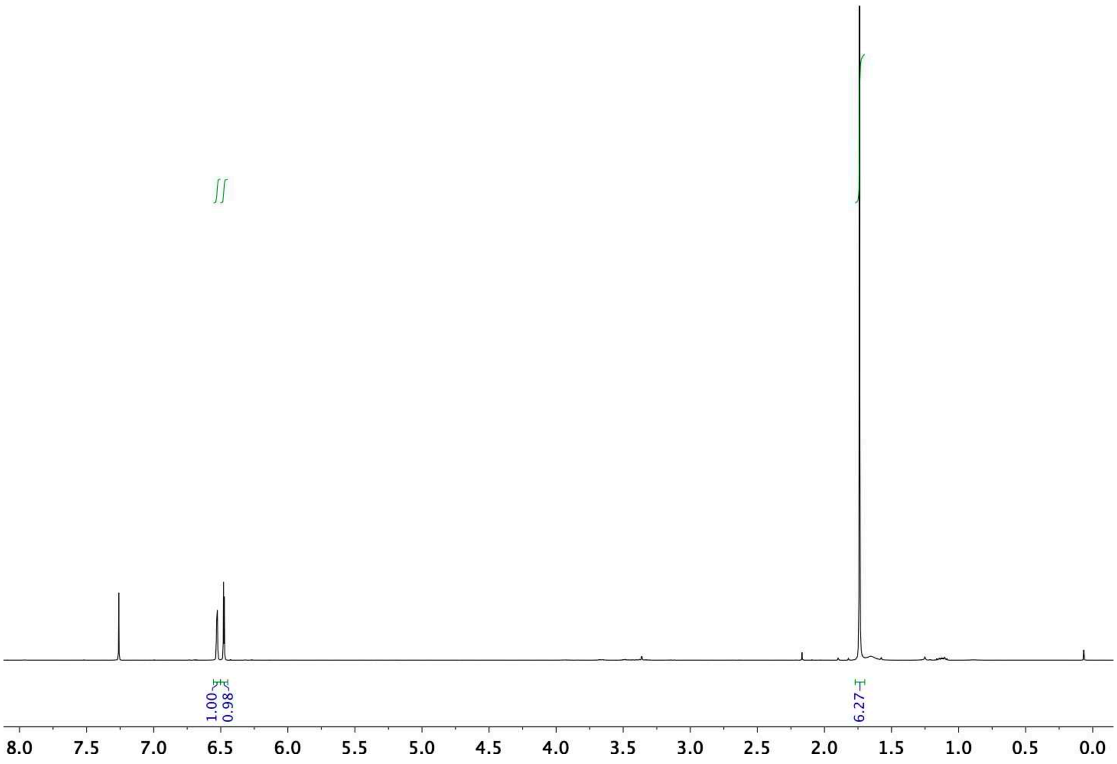

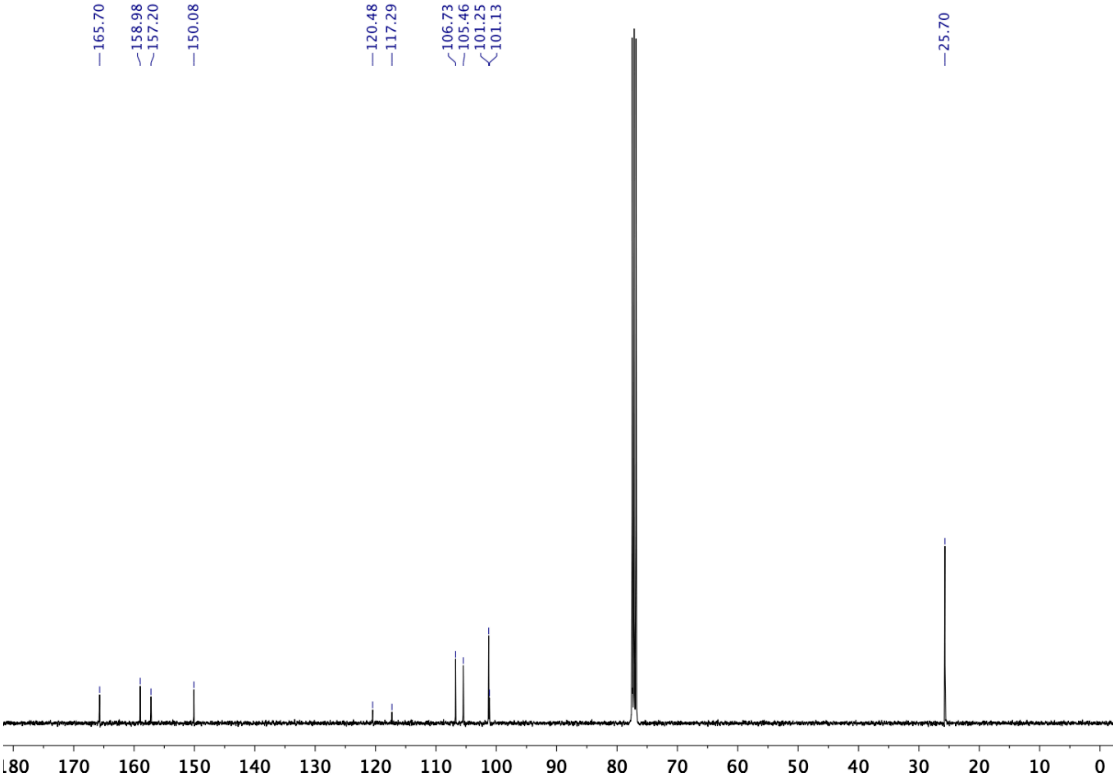

7-methoxy-2,2-dimethyl-5-(4,4,5,5-tetramethyl-1,3,2-dioxaborolan-2-yl)-4H-benzo[d][1,3]-dioxin-4-one–D_3 ***(4b)***

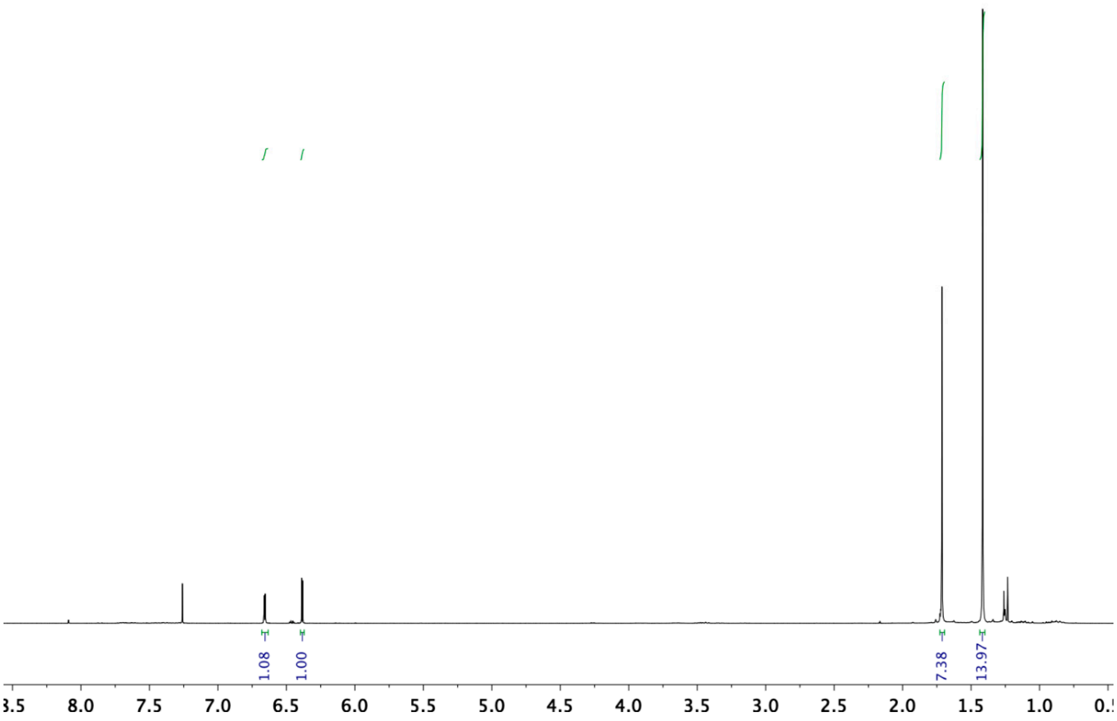

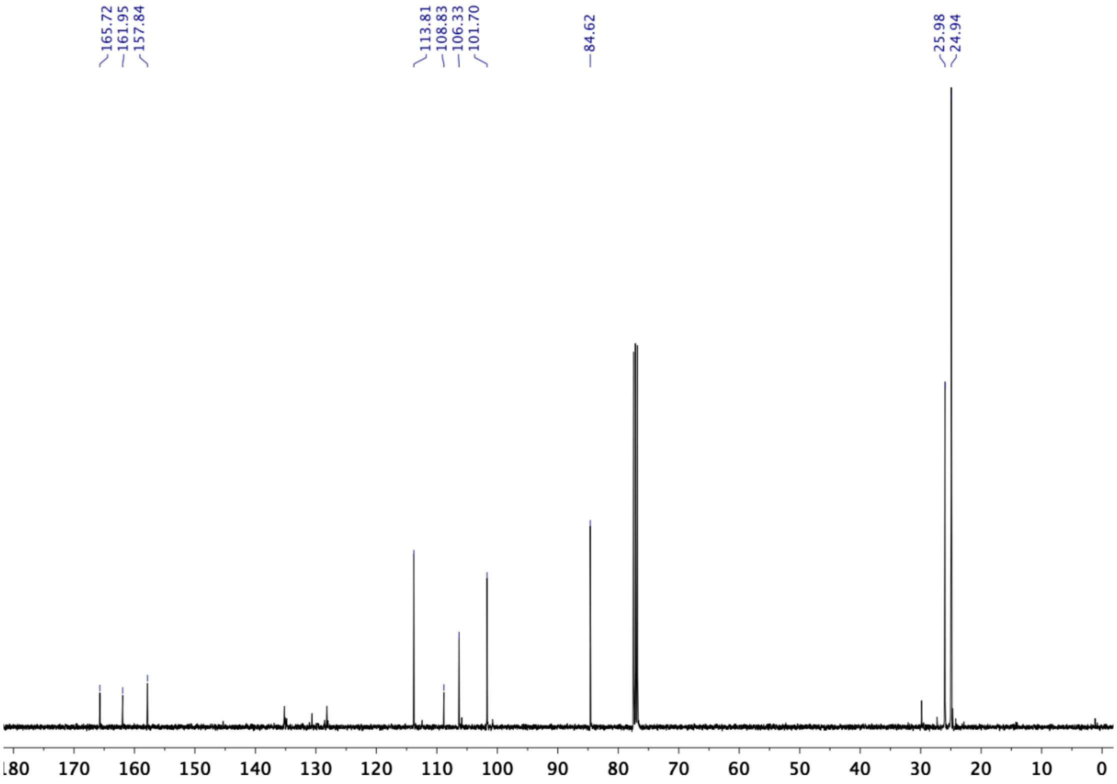

*5-((2S,3R,4aR,6R,8aR)-2,3,4a,5,6,8a-hexahydro-6-hydroxy-2,3-dimethoxy-2,3,6-trimethylbenzo[b][1,4]dioxin-7-yl)-7-methoxy-2,2-dimethyl-4H-benzo[d][1,3]dioxin-4-one-D3 (**11b**)*

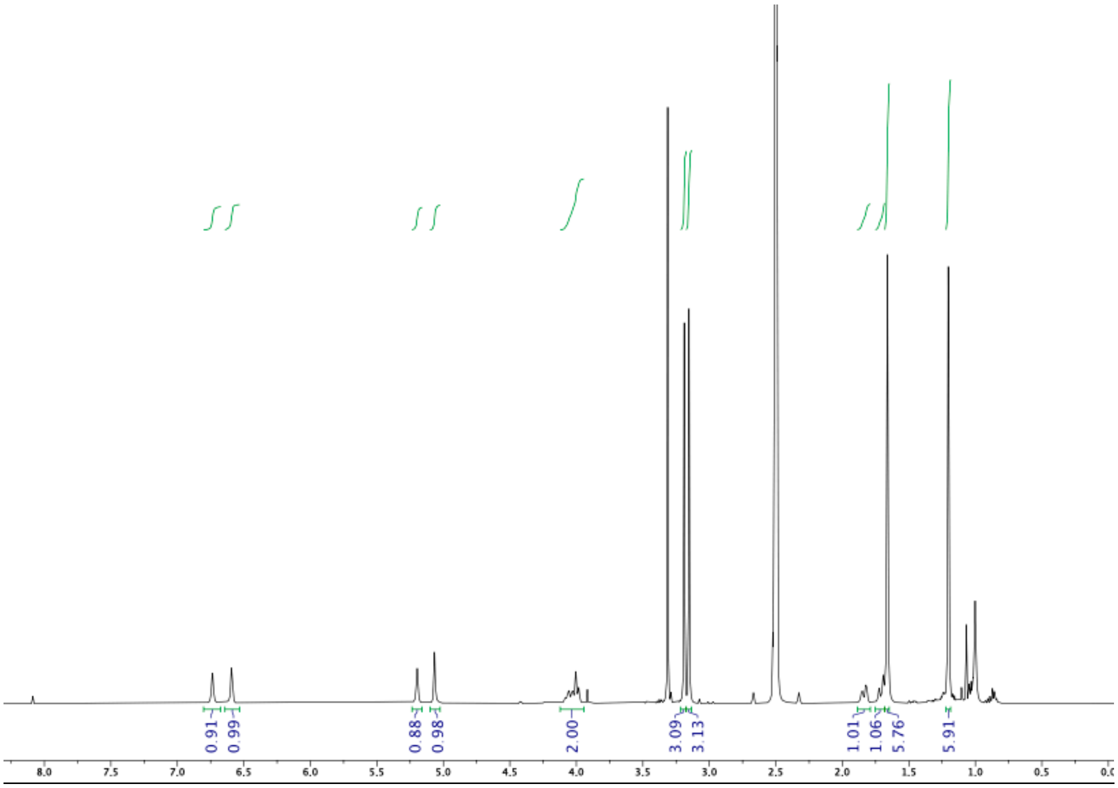

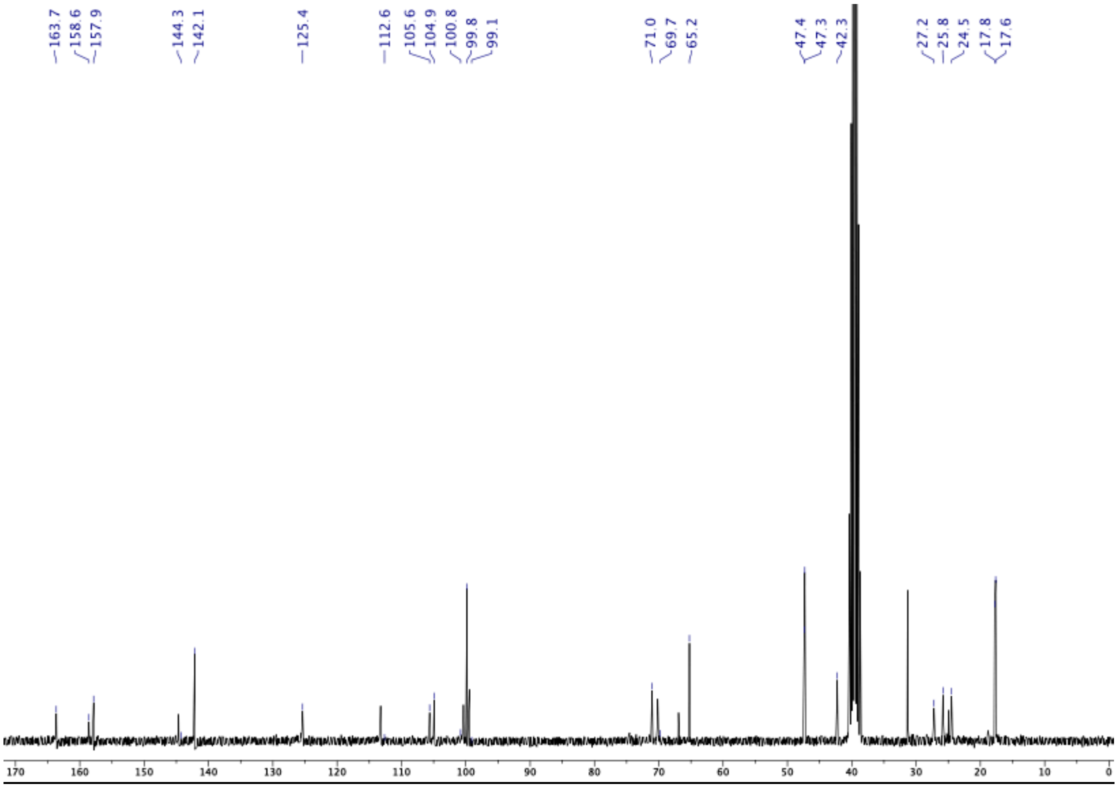

(6aR,7aR,9S,10S,11aR)-4-Hydroxy-2,9,10-trimethoxy-7a,9,10-trimethyl-6a,7,7a,9,10,11a-hexahydro-5H-benzo[c][1,4]dioxino[2,3-g]-chromen-5-one-D₃ (***12b***)

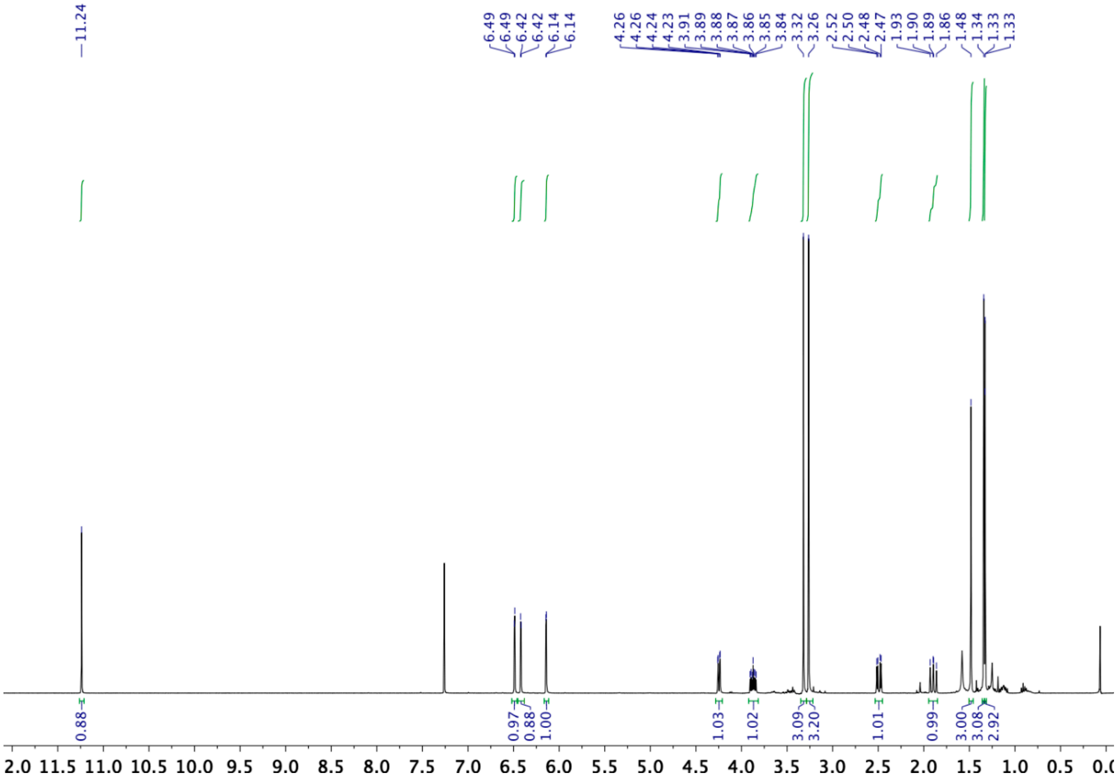

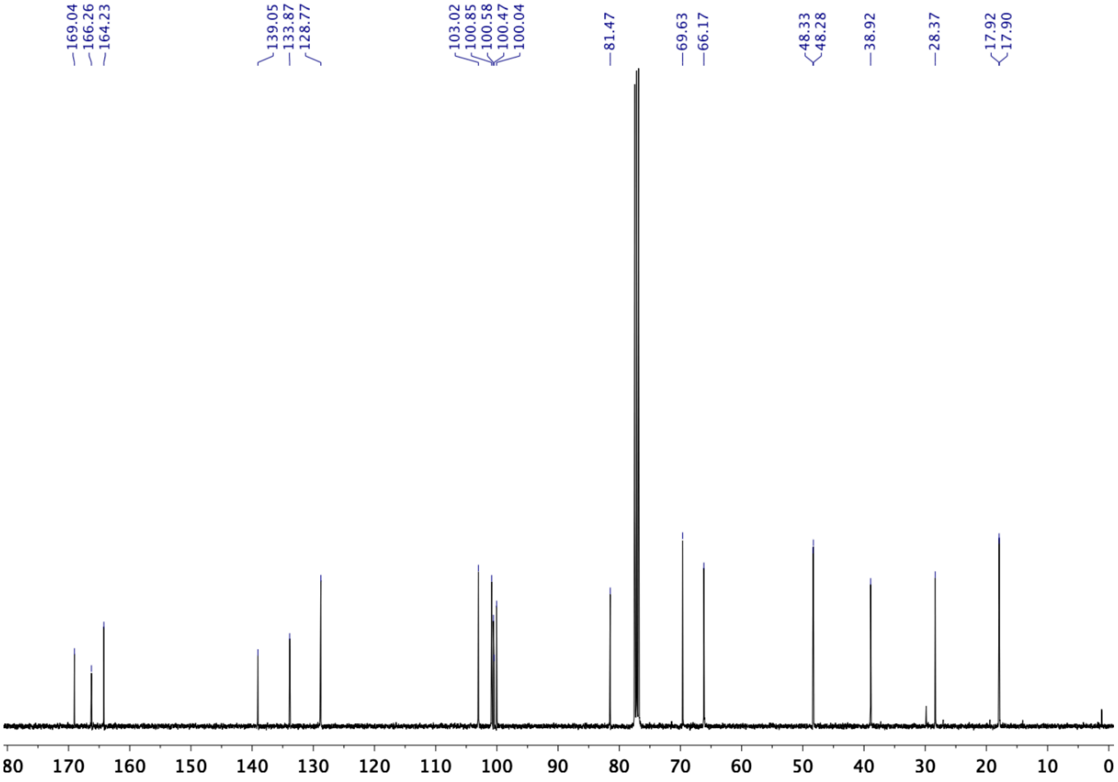

(2R,3R,4aR)-2,3,4,4a-tetrahydro-2,3,7-trihydroxy-9-methoxy-4a-methylbenzo[c]chromen-6-one-D$_3$
*((−)-Altenuene-D$_3$, **1b**)*

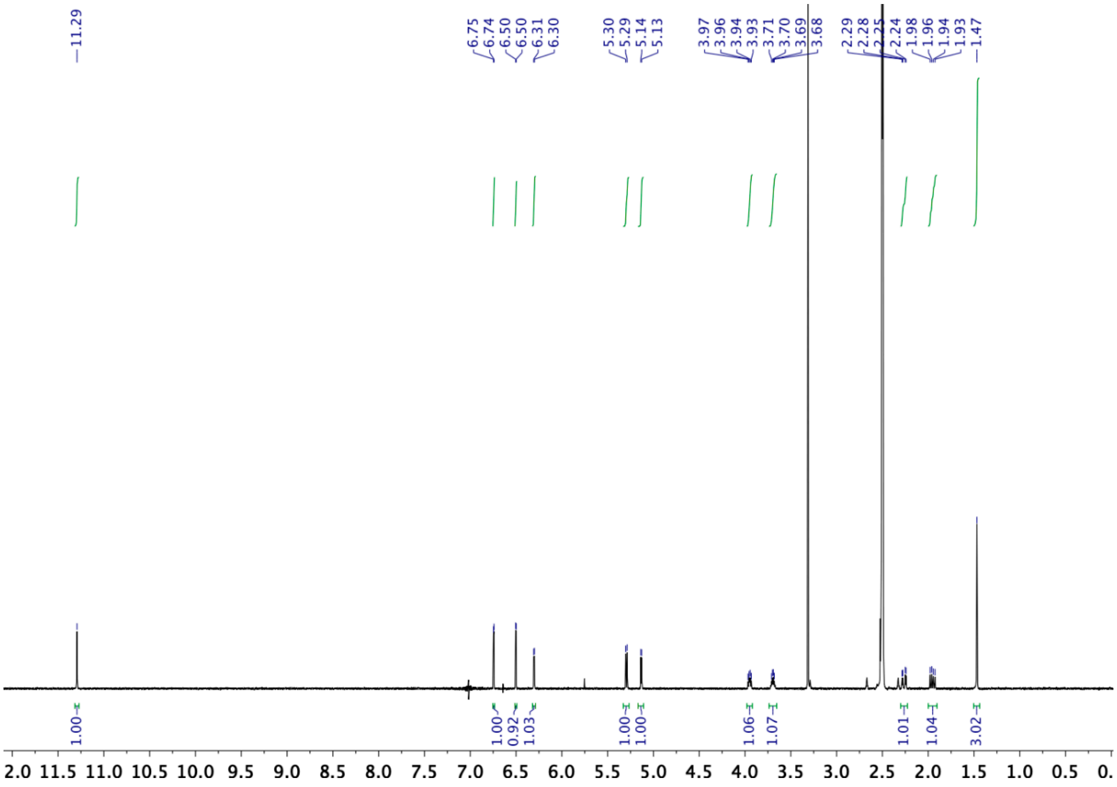

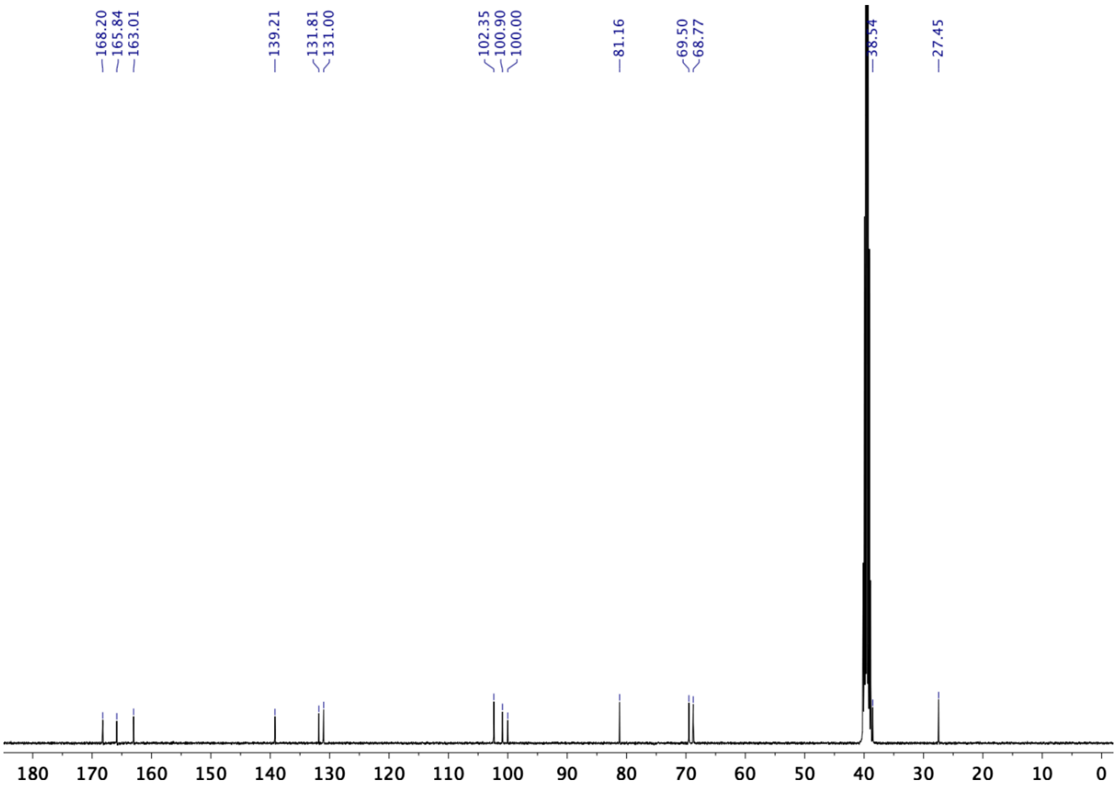

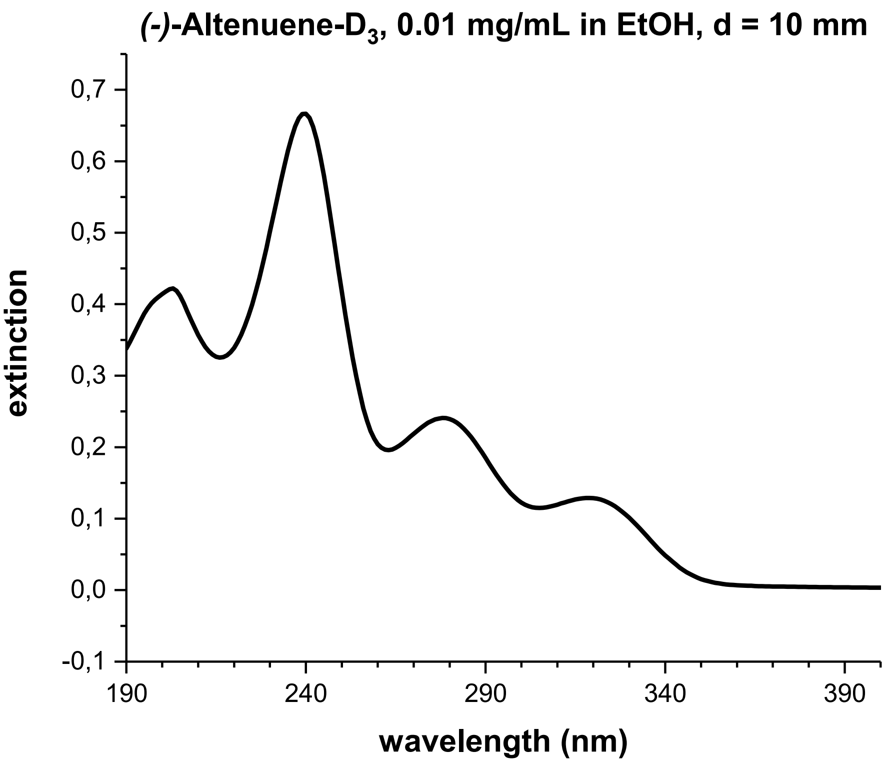
(-)-Altenuene-D3, 0.01 mg/mL in EtOH, d = 10 mm
extinction
0,7
0,6
0,5
0,4
0,3
0,2
0,1
0,0
-0,1
190
240
290
340
390
wavelength (nm)

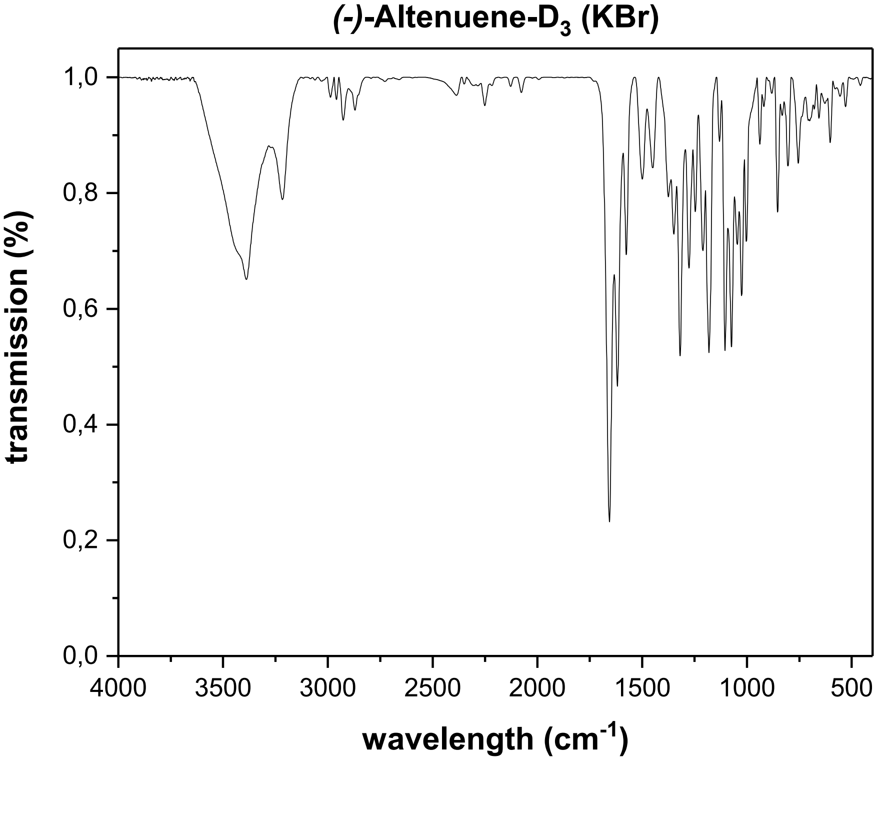
(-)-Altenuene-D3 (KBr)
transmission (%)
1,0
0,8
0,6
0,4
0,2
0,0
4000
3500
3000
2500
2000
1500
1000
500
wavelength (cm-1)

*(2R,3R,4aR)-2,3,4,4a-tetrahydro-2,3,7-trihydroxy-9-methoxy-4a-methylbenzo[c]chromen-6-one ((−)-Altenuene, **1a**)*

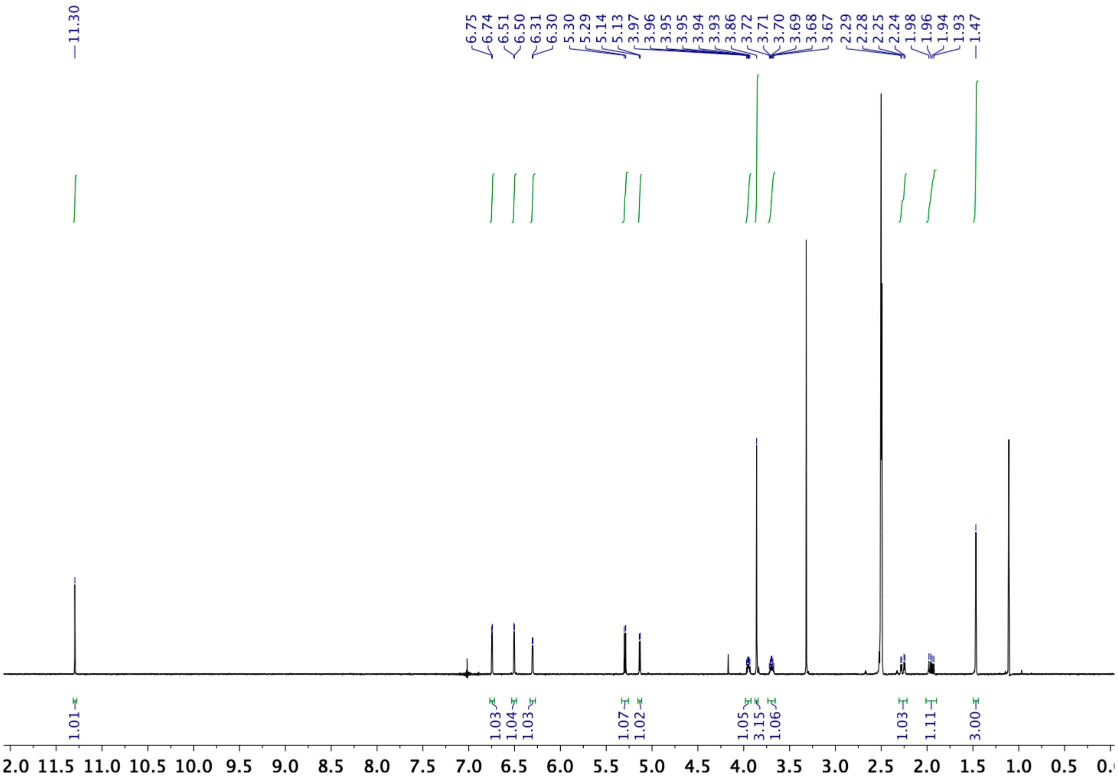

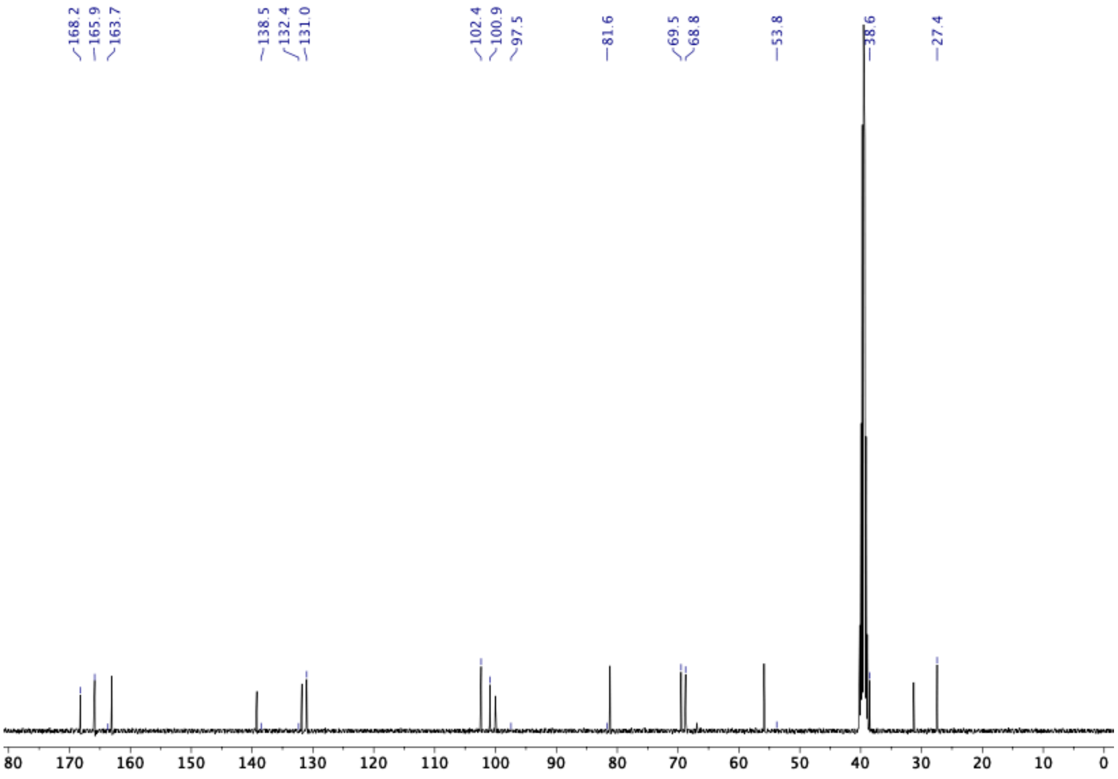

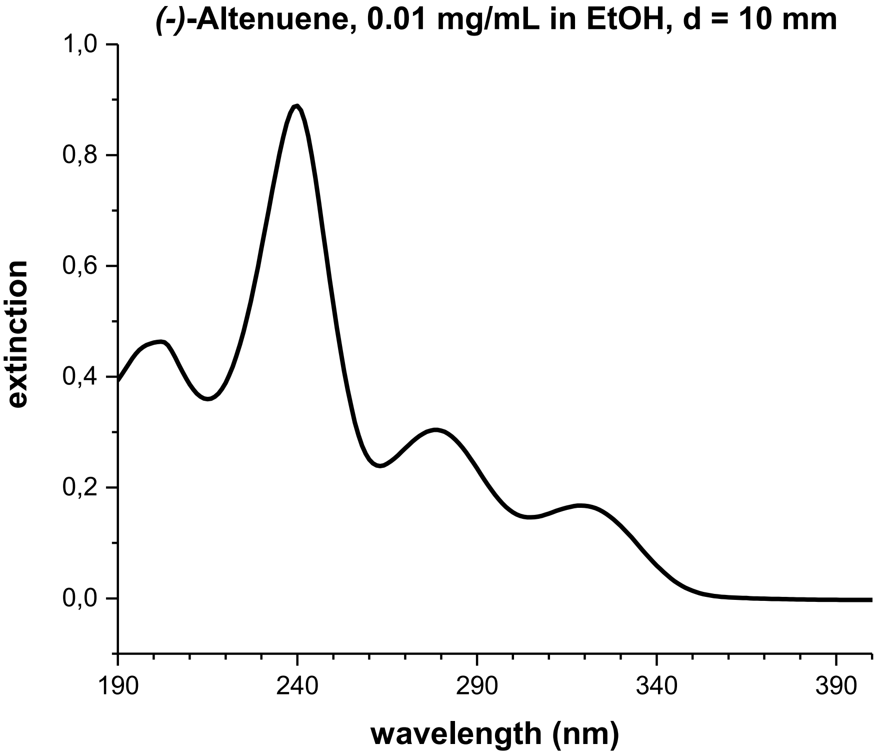
(-)-Altenuene, 0.01 mg/mL in EtOH, d = 10 mm
extinction
1,0
0,8
0,6
0,4
0,2
0,0
190
240
290
340
390
wavelength (nm)

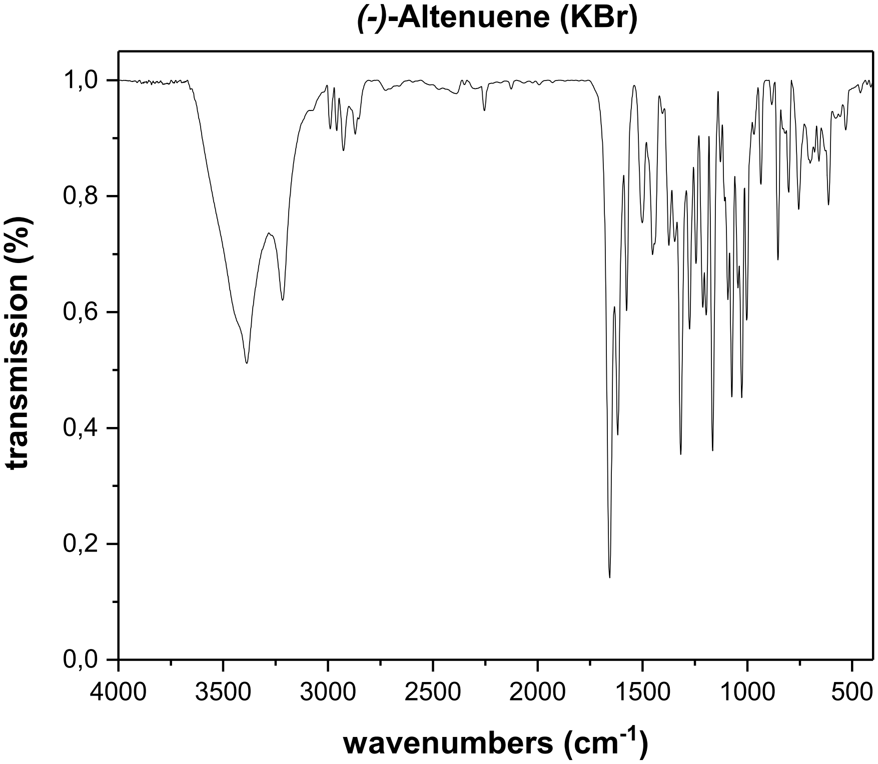
(-)-Altenuene (KBr)
transmission (%)
1,0
0,8
0,6
0,4
0,2
0,0
4000
3500
3000
2500
2000
1500
1000
500
wavenumbers (cm-1)
